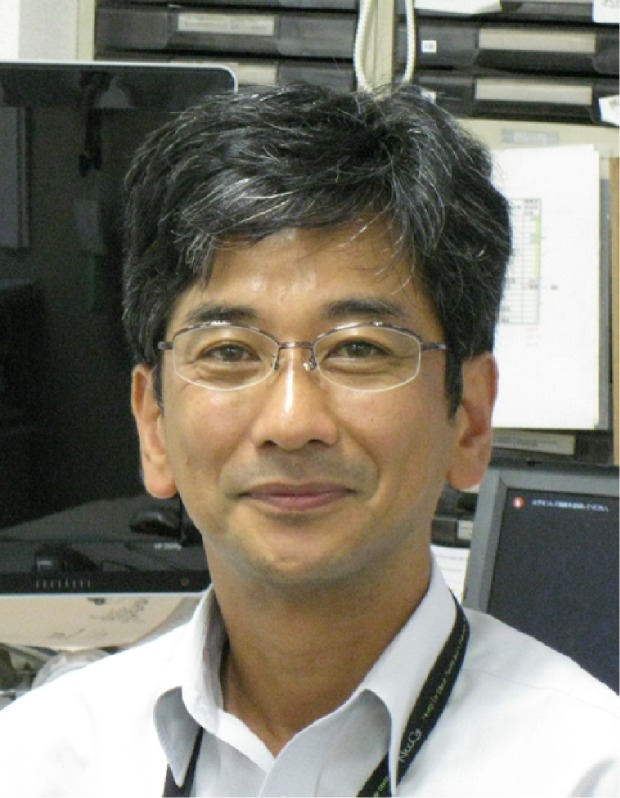# Message From the New Editor-in-Chief

**DOI:** 10.2188/jea.JE20130031

**Published:** 2013-05-05

**Authors:** Tomotaka Sobue

It is my great privilege to take over as Editor-in-Chief (EIC) of the *Journal of Epidemiology* from Dr. Hiroyasu Iso, who served in that capacity in 2011–2012. Dr. Iso was elected Executive Head of the JEA, and it is almost impossible to manage simultaneously the responsibilities of these important jobs. I was the EIC in 2008–2010, so this is my second opportunity. Despite his relatively short tenure, Dr. Iso has made great contributions to the journal, such as promoting publication of review articles, particularly from authors outside Japan. In addition, the number of manuscripts from outside Japan has notably increased: in 2012 the percentage of articles submitted, by country/region, was 32% from Japan, 14% from China, 10% from Taiwan, 8% from Korea, 12% from other Asian countries (including Australia), 10% from Europe, 9% from the Middle East, 3% from the United States, and 1% each from South America and Africa. The average impact factor in 2011 was 1.858. To manage the increase in articles submitted to the journal, the number of associate editors on the editorial team was increased from 18 to 25, which will ensure that the editorial process will continue in a timely fashion. Editorial board members are now expected to understand both the most recent research in their fields and the processes and priorities of journal management, such as how to address plagiarism, fabrication, and falsification. The time may come when the tasks of journal management exceed the resources of our academic society, but at present we have decided to continue to manage the journal internally, so as to preserve our independence. The editorial team aims to maintain the high standards of an international journal by continuing to publish original and review articles on a wide range of research topics and to ultimately establish our journal as a leader in the field of epidemiology.

 Tomotaka Sobue, MD, MPH Editor-in-Chief Journal of Epidemiology  Professor of Environmental Medicine and Population Sciences Department of Environment and Social Medicine Osaka University Graduate School of Medicine

**Figure:**